# Medical Opinion and Movement

**Published:** 1910-03-12

**Authors:** 


### Medical Opinion and Movement.

### Traumatic Lesions.

Cicatrices adherent to the osseous margin of the orbit not infrequently occur as a result of trauma, suppuration, and the like. When such lesions lead to deviations of the eyelids or other consequences of a serious nature some plastic operation may be necessary, but the condition is often of a more trivial nature involving only æsthetic considerations, and for such cases Dr. Axenfeld proposes instead of paraffin injections the transplantation of a fragment of adipose tissue removed from the subcutaneous tissues of the abdomen. Under general anæsthesia a small incision is made below the adherent area, and the latter is carefully separated from the underlying bone, so that the cicatrix can be raised up to the level of the surrounding skin. An adipose graft of suitable size and shape is removed from under the skin of the abdomen and introduced through the buttonhole incision, which is then sutured. After the operation the skin is generally red, slightly tense, and raised above the surrounding surface, but this soon subsides. Dr. Axenfeld has carried out the operation in three cases, and according to the "before and after" photographs reproduced in the *Klinische Monatsblätter für Augenheilkunde* the results are extremely satisfactory.

### Hæmostatics and Blood Coagulation.

Dr. Ciuffini has made some interesting investigations on the effect of certain reputed hæmostatics upon the coagulation of the blood. He finds that subcutaneous injections of gelatine that has not been previously sterilised have a decided coagulative power upon the blood both by accelerating the process and also by increasing its extent. But sterilisation by heating gelatine to 130° for half an hour completely destroys this property to coagulate. Given by the mouth in 10-per-cent. solution the gelatine did not raise the coagulability to any appreciable extent, and the same may be said of rectal injections. Chloride of calcium administered in doses amounting to 2 or 3 grammes in 24 hours, also showed little or no effect upon the coagulability of the blood. On the other hand, perchloride of iron had a prompt and definite effect, although less intense than the unsterilised gelatine injections. Ammoniated citrate of iron is quite inactive, even when injected subcutaneously. Ipecacuanha, which has been especially advocated by Trousseau in severe hæmoptysis, is found to cause some small increase in coagulability, but this passes off within 24 hours. It would appear to exercise a hæmostatic effect rather in this way than by a fall in blood pressure, as observations showed actually a slight increase of arterial tension from 108 to 113 mm.

From these observations it appears that gelatine and perchloride of iron show the best hæmostatic properties. But with injections of unsterilised gelatine there is a serious risk of tetanus as well as other less virulent infections, and sterilisation renders it useless. Iron perchloride cannot be given in any considerable amount without setting up gastric irritation. In view of these difficulties the author has experimented on dogs with a sort of artificial gelatine consisting of a mixture of a concentrated solution of gum arabic and perchloride of iron. The two substances are sterilised separately, and the mixture is injected subcutaneously after warming sufficiently to liquefy it. Dr. Ciuffini finds that the mixture in question has a considerable coagulative power on the blood. Its action is superior to all other hæmostatics except unsterilised gelatine injected subcutaneously. The effect, moreover, is very persistent, remaining the same for 24 hours, whereas the action of gelatine begins to diminish at that interval of time. According to this author the hæmostatic effects of drugs usually employed for this purpose are so feeble in their action that they can be of little or no account to arrest a severe hæmorrhage or combat a hæmorrhagic diathesis.

This curious fact brought out by Dr. Ciuffini of the nullifying effect of sterilisation upon the coagulative power of gelatine is especially interesting in relation to the past history of gelatine injections. It may be remembered that Lancereaux first advocated subcutaneous injections of gelatine solution for the treatment of saccular aneurysms, and published a number of cases in which the treatment appeared to have resulted in a deposit of fibrinous clot within the sacs. The treatment was tried at one of the London hospitals, and gave some favourable results, but was abruptly arrested by the occurrence of two fatal cases of tetanus poisoning owing to imperfect sterilisation of the gelatine. Subsequently the injections were tried again in a series of cases, special precautions being taken for thorough sterilisation, and in this series the results were entirely negative, and no improvement occurred from the treatment.

### Uric Acid and the Bacillus Coli.

Some interesting research has been carried out by Dr. Holgar Trautner, by which he claims to show a close causal relationship between the formation of uric acid in the system and the activity of the bacillus coli in the intestines. His early observations showed that in those affected with gout constipation caused a marked increase in the uric acid excreted, whatever the diet might be. Following this idea he turned his attention to the presence of uric acid in the urine of infants. According to his observations uric acid is absent in very young infants in a healthy condition, and present in only minute quantities in older infants. On the other hand, in infants with digestive troubles, especially diarrhœa, in whom the presence of the bacillus coli in the fæces was ascertained by the indol reaction, there was almost always an appreciable quantity of uric acid in the urine. These observations extended over 25 infants, 12 healthy and 13 suffering from digestive troubles, and they showed a close parallelism between the amount of uric acid excreted and the presence of the bacillus coli as shown by the indol reaction. Experiments on young dogs confirmed these results. Five dogs were infected with milk containing the bacillus coli, and out of 24 urinary analyses eight showed the presence of uric acid. No uric acid was found previously, and ordinary milk failed to produce it. Finally, the author finds, as has already been observed by others, that in typhoid fever there is a marked diminution in uric acid excretion. In 32 cases he has watched the uric acid steadily diminish with the progress of the disease, and even during convalescence it remains considerably below normal. In 327 control individuals the uric acid was always in excess of that found in any of the typhoid cases. Dr. Trautner explains these results by the fact that the typhoid bacillus destroys the bacillus coli in the intestine, and hence the diminution of uric acid, which results from the activity of the latter micro-organism. Clinical experience so far conforms with this view that one of the fundamental points in the treatment of gout is to keep the bowels acting very freely.

### The Premonitory Signs of Eclampsia.

The early symptoms and signs of impending eclampsia are described in the *Glasgow Medical Journal* by Dr. Logan, who points out that these have attracted much more attention abroad and in America than in Britain. In a large proportion of cases, though not of course in all, inquiry reveals that before they culminate in convulsions or coma the patients exhibit suspicious signs for days or even weeks beforehand, and by proper treatment the onset of the later and more serious symptoms can be postponed or altogether avoided. One of the most constant of these premonitory symptoms is headache, which may be of varied character, and is often accompanied by giddiness, nausea, and disturbances of vision such as flashes of light, muscæ volitantes, or even blindness. Photophobia, diplopia, and strabismus are also sometimes noticed. Loss of memory and temporary aphasia are not unknown just before fits begin. Epigastric pain, paroxysmal or constant, is another significant symptom: it may be accompanied by nausea and bilious vomiting. Jaundice is occasionally seen, and with it enlargement of the liver, which may also be tender: this is said to be of especially bad prognostic import. Œdema, wherever occurring, is highly significant, and so is a high pulse tension: decrease of urea secreted is equally valuable as a sign of impending eclampsia. All these are subsidiary in importance to the discovery of albuminuria. If the urine is scanty and high coloured, with albumin and casts, and if any other of the suspicious symptoms is present, prompt measures of prophylaxis must be adopted.

First stand the usual rules of hygiene, which are but too often totally neglected during pregnancy. Cleanliness, baths, exercise, fresh air, and the rest of everyday personal hygiene are more imperative at this time even than at any other. When in any given case it seems likely or possible that the premonitory symptoms of eclampsia are being exhibited the patient should be ordered to bed at once, and as the symptoms decrease great care should be taken not to allow any resumption of ordinary life except by very gradual steps. Alcohol should be entirely prohibited, and very little nitrogenous food of any sort should be allowed. Milk must constitute the staple of diet in all such cases, but if it proves irksome, and the other circumstances of the case allow, in ten days or so bread-and-butter, milk puddings, tea, and fruit may be added to the diet. An abundance of fluids, such as lemonade, water, etc., should be given to promote diuresis. At the same time attention is to be paid to the skin, and its activity stimulated by tepid baths, friction, and massage, and in urgent cases hot baths or hot packs. The drugs which have been proved to be of value form but a very small list. Calomel is the best intestinal antiseptic and aperient, and may be freely given. Simple diaphoretics and diuretics such as potassium bicarbonate and acetate, or liquor ammoniæ acetatis, are unobjectionable. Alkalies given by subcutaneous transfusion have their advocates. Pilocarpine ought to be discarded altogether. Morphine has several fervid advocates, and has been combined with full doses of thyroid extract, in which some practitioners of experience have firm faith. Saline irrigation of the large bowel is mentioned with approbation, potassium bicarbonate being added to the normal salt solution used.

### Inoperable Sarcoma.

The treatment of inoperable sarcomata by means of the mixture of toxins from Bacillus prodigiosus and other organisms, which is usually known by the name of its inventor as Coley's fluid, has never been very successful in this country. Many surgeons have tried it, but very few have been satisfied with the results obtained. The reason is probably the somewhat too high estimate of the value of this vaccine which was at first set upon it by its discoverer, and another may be the successive modifications which have displaced the original method of preparing the fluid. Dr. Coley now claims that 11 per cent. of inoperable sarcomata disappear entirely under his treatment. These claims have been reviewed in the light of his own experience and that of colleagues by Dr. Loeb, who publishes his independent conclusions in the *Journal of the American Medical Association.* He finds the percentage of cures to be, approximately, something more than four, but less than nine: even this is a remarkable thing when it is remembered for what hopeless cases the treatment is alone recommended. He further believes that as a post-operative procedure it may diminish the likelihood of recurrence, and that in certain cases it may limit the need for amputation in sarcomata of some of the long bones. He confesses ignorance of how the action is obtained, but believes that either the toxins or their effect on the general metabolism have an injurious influence on sarcoma cells.

### A New Reflex in Meningitis.

In May 1908 attention was called in these columns to the work of Brudzinski with reference to certain reflexes discovered by him in children suffering from tuberculous and epidemic meningitis. In such cases when passive flexion is made of the lower limb of one side, an analogous movement of the corresponding side is seen. This the author called "the identical contralateral reflex." In some cases, however, the movement of the corresponding limb is one of extension, and not of flexion. This the author termed "the reciprocal contralateral reflex." As the result of further research Brudzinski has noticed another reflex in meningitis, which he terms the sign of the neck. Passive forward flexion of the neck causes flexion of the knee and hip-joints, the limbs being flexed on the pelvis, sometimes to a marked extent. In meningitis, whether tuberculous, cerebro-spinal, serous, or pneumococcic, the neck sign was found in 97 per cent., Babinski's sign in 50 per cent., Kernig's sign in 57 per cent., and the contralateral reflexes in 66 per cent. of cases. The neck sign was found wanting sometimes in the stages preceding the death agony. The phenomenon is difficult to explain, but probably the most important part is played by a muscular hypertonus of the lower limbs, and a physiological predominance of the extensors of the neck and back over the flexors of the lower limbs. On account of its pathognomonic significance, its constancy, and its simple application it would seem that this sign is worthy of being better known, and of being systematically studied in all suspected cases of meningitis.

### Menstruation during Pregnancy.

It is an obstetrical axiom that all menstrual loss during pregnancy is modified as regards duration, quantity, or quality. The following observation reported by M. E. Vogt in the *Zentralblatt für Gynäkologie* may perhaps supply the exception which proves the rule, and is interesting because of the familial character of the phenomenon. The subject was a young woman of 26. Menstruation had commenced at the age of 12, lasted from 5 to 8 days, was normal in amount, and painless. At the age of 22 she was confined for the first time, the birth being a normal one. Since that time she had had no miscarriage. Her general health was very good and her past history uneventful. When seen at term she declared that she had menstruated up to the sixth month of pregnancy, without any irregularity of date, length, or abundance of the catamenia. Menstruation became re-established eight weeks after the confinement. Surprised by this history the author made an inquiry into the history of the family, which was of Tzech origin. He learnt that the twin sister of his patient had likewise menstruated up to the sixth month of her only pregnancy. A younger sister, seven months pregnant at the time, had menstruated four weeks previously. The mother of the family declared that in every case she herself menstruated up to the sixth month of pregnancies; and when her daughters left her to get married she had been in the habit of warning them of this family peculiarity.

### The Leuko-diagnosis of Cancer.

At a meeting of the Société de Biologie, Messrs. Achard, Bénard, and Gagneux reported that they had obtained *in vitro* a specific reaction in cases of cancer by the action of extracts of mammary epitheliomata on leucocytes. The activity of the white cells of cancerous subjects, when treated in this way, is found to be hardly diminished as a rule, and in some cases even to be increased. The cells of healthy subjects, on the other hand, show a markedly enfeebled activity. This rule has been verified with regard to the extracts made from 29 different tumours obtained from various parts of the body. The reaction failed in one case of osteosarcoma, in one case of mammary cancer operated on a month and a half previously, and in one case of uterine cancer treated for seven months by radium and clinically cured. Two cases in which the reaction was negative are of peculiar interest from the point of view of diagnosis. In one, the question of lingual cancer had been raised, but histological examination showed that the condition present was interstitial glossitis. In the other diagnosis lay between ulcer and cancer of the stomach, operation subsequently revealing the presence of an ulcer. Extracts prepared with different tumours of the same nature do not always give the same result in any one case. Thus a cancer of the testicle gave a positive reaction to one extract and a negative reaction to another. These facts, in the opinion of the authors, show that it is possible to draw valuable conclusions from specific leuco-reactions as to the diagnosis of cancer.

### Syphilitic Pleurisy.

At a recent meeting of la Société Médicale des Hôpitaux, Drs. Roger and Sabaréanu reported the case of a woman of 38 who was admitted to hospital for a serous pleuritic effusion of lymphocytic type. The patient, who had been treated previously for tuberculosis, gave a history of having contracted syphilis at the age of 22, and of having for some years been troubled with gummatous manifestations. Tuberculous pleurisy was, however, suspected, despite the fact that no albumin could be discovered in the sputum, and that inoculation into the guineapig was negative. On the other hand, Wassermann's reaction to the pleuritic fluid was positive, and under mercurial treatment the pleuritic effusion was absorbed rapidly. The authors, with the object of finding out whether the presence of Wassermann's reaction in the fluid from an effusion occurring in a syphilitic was sufficient by itself to prove the syphilitic nature of the disease, have experimented on nine other persons suffering from syphilis, by seeking for the deviation of the complement in serous fluid obtained from these persons by blistering. In eight of these cases the result was positive, the only case in which it was negative being that of a subject who was at the time undergoing vigorous mercurial treatment. From these observations the authors are led to conclude that any serous effusion occurring in a syphilitic can deviate the complement without on that account being of a syphilitic nature.

